# Effect of blueberry extract on enhancement of radiosensitivity in non-Hodgkin lymphoma by modulating proliferation and apoptosis

**DOI:** 10.1007/s40199-025-00577-8

**Published:** 2025-10-03

**Authors:** Neda Moradloo, Samaneh Arab, Hamed Rezaee Jam, Samira Asgharzadeh, Saeed Shokri, Leila Nasehi, Ali Nokhodchi

**Affiliations:** 1 Student Research Committee, Department of Medical Laboratory Sciences, School of Allied Medical Sciences, Zanjan University of Medical Sciences, Zanjan, Iran; 2 Department of Tissue Engineering and Applied Cell Sciences, School of Medicine, Semnan University of Medical Sciences, Semnan, Iran; 3 Department of Radiology Technology, School of Allied Medical Sciences, Zanjan University of Medical Sciences, Zanjan, Iran; 4 Cellular and Molecular Research Center, Basic Health Sciences Institute, Shahrekord University of Medical Sciences, Shahrekord, Iran; 5 Anatomy and Histology, School of Medical Sciences, Faculty of Medicine and Health, University of Sydney, Sydney, Australia; 6 Cancer Gene Therapy Research Center, Zanjan University of Medical Sciences, Zanjan, Iran; 7 Pharmaceutics Research Laboratory, School of Life Sciences, University of Sussex, Brighton, UK

**Keywords:** Burkitt lymphoma, Non-Hodgkin, Blueberry extract, Radiation-sensitizing

## Abstract

**Background:**

Non-Hodgkin lymphoma, a major cancer type, is usually treated with radiotherapy but encounters challenges with resistance and toxicity. Therefore, the treatment of non-Hodgkin lymphoma needs agents to be very effective while protecting healthy cells. Blueberry extract, rich in micronutrients, flavonoids, and bioactive compounds, may inhibit cancer cell growth and induce apoptosis without harming normal cells.

**Objectives:**

This study investigates the efficacy of blueberry extract in combination with radiotherapy as a radiosensitizer on Raji cells, a model for highly invasive non-Hodgkin lymphoma.

**Methods:**

First, Raji cells were treated with blueberry extract alone and in combination with a single dose of 2 Gy radiotherapy. The effects of blueberry extract on inhibiting proliferation and induction of apoptosis in Raji cells were investigated by MTT assay, flow cytometry (Annexin-V-FITC), cell cycle analysis, and quantitative gene expression analysis of *BAX*,* BCL-2* and *XPA*. Its role in improving the efficacy of radiotherapy on cancer cells was also investigated.

**Results:**

Treated cells with blueberry extract alone and in combination with radiotherapy showed reduced viability, increased induction of apoptosis and a higher proportion of cells in the SUB-G1 cell cycle phase was detected. Additionally, gene expression analysis indicated upregulation of the pro-apoptotic gene *BAX* expression and decreased anti-apoptotic gene *BCL-2* expression, along with elevated expression of *XPA* as an indicator of DNA damage after radiotherapy.

**Conclusion:**

The study suggests that blueberry extract stimulates apoptosis in Raji cells and could serve as an anti-cancer drug. Furthermore, the combination of this extract with radiotherapy could be used as a radiosensitizer.

**Graphical Abstract:**

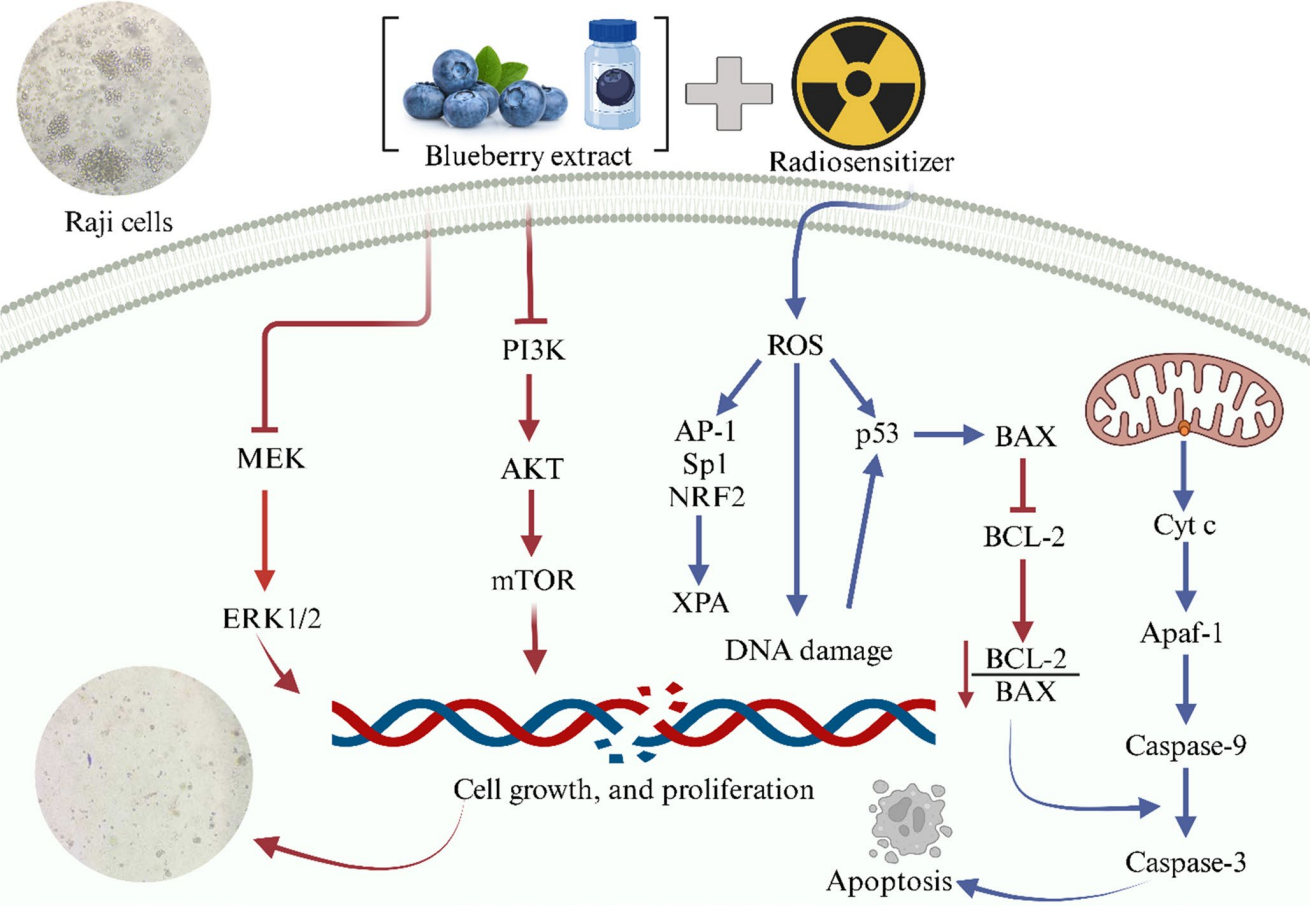

## Introduction

Non-Hodgkin lymphoma (NHL) is a heterogeneous group of hematologic malignancies that account for an estimated 90% of all lymphomas. In 2020, it was the 11th most common cancer and contributed to 2.6% of all cancer-related mortality [1, 2]. Depending on the type of NHL, treatment can include chemotherapy, radiotherapy, stem cell transplantation, targeted therapy, and immunotherapy [2–5]. Radiotherapy is one of the most important cancer treatments and is considered a cost-effective therapeutic option [6]. However, resistance to radiotherapy and its toxic effects on healthy cells have limited its use [6, 7]. Therefore, nowadays, the use of agents that have synergistic effects on radiotherapy and increase the sensitivity of radiotherapy without being toxic to healthy cells is now emphasized [8].

Although radiosensitizers are under development, current results do not fully meet clinical requirements, and concerns remain regarding potential damage to normal cells following radiotherapy when applied systemically [8, 9]. A radiosensitizer not only enhances sensitivity to radiotherapy but also selectively targets cancer cells without damaging normal tissue which is crucial [8].

Nowadays, there is a growing tendency to use herbal medicines to treat various diseases because they are safe, non-toxic, and easily accessible [8, 10]. One type of herbal medicine is blueberry. Blueberries are a rich source of essential nutrients, including sugars, vitamins, folic acid, minerals and organic acids. They also contain bioactive compounds such as flavonoids, which include flavonols, quercetin, myricetin, kaempferol, catechin, epicatechin, gallocatechin and anthocyanins [11, 12]. Polyphenols present in blueberry extract (BE) and other herbal medicines reduce cancer cell proliferation through the inhibition of the PI3K/Akt/mTOR pathway [13, 14]. As a result, recent studies suggest blueberry extract as a potential anti-cancer and anti-inflammatory agent [15, 16]. Several studies have reported that in various types of cancers such as lung, breast, liver and melanoma, extraction of blueberry or its components can inhibit growth and induce apoptosis while not affecting normal cells [16–19]. Treatment of hepatocellular carcinoma with this extract increased the expression of BCL2-associated X protein (*BAX)* and a reduction in the expression of B-cell lymphoma 2 (BCL-*2)* protein, which played a role in promoting apoptosis induced by this extract [20]. In addition, several studies have shown that combined treatment with herbal drugs such as blueberry extract, curcumin, and myricetin can increase the sensitivity of cancer cells to radiotherapy [8, 17, 21, 22]. Therefore, it was interesting to investigate the toxicity and radiosensitizing properties of this extract in other types of cancer cells.

In this study, the Raji cell lines, known for its highly invasive nature among non-Hodgkin lymphomas, were utilized to examine the combined effect of blueberry extract and radiotherapy on increasing cancer cell sensitivity to radiation and promoting apoptosis. This research aims to offer a novel perspective on the anticancer and radiosensitizing properties of blueberry extract on Burkitt lymphoma cells.

## Materials and methods

### Materials

Penicillin/streptomycin was obtained from Gibco in the USA. DMSO and MTT powder were also purchased from Sigma (Germany). Additionally, phosphate-buffered saline (PBS) was acquired from Gibco in the USA. Trypan blue dye was acquired from Merck (Germany).

## Cell culture

The Raji cell lines (ATCC^®^ CCL-86™), were cultured in RPMI media (Gibco, USA) with 1% penicillin/streptomycin (100 µg/mL; Gibco, USA), enriched by 10% FBS (Gibco, USA), at 37 °C in a standard cell culture incubator. The cell Lines were a gift from the Tehran University of Medical Sciences, Iran. Trypan blue dye staining was used to determine the vitality of the cells, and cultures showing more than 90% viability were chosen for further studies [23].

## Experimental treatment

An X-ray linear accelerator was used to perform experimental irradiation (IR) at room temperature with a dose rate of 2.0 Gy/min. Blueberry extract, a gift from Shahrekord University of Medical Sciences, Iran, was used in in vitro experiments to pretreat cultures one hour before irradiation [8]. Raji cells were treated with blueberry extract dissolved in RPMI-1640 at doses ranging from 0 to 1000 µg/mL (stored at -20 °C until use). Cell treatments were administered over a period of 24, 48, and 72 h [17, 22].

## Cell viability assay

The IC50 level and cell viability were assessed using the MTT (3-(4,5-dimethylthiazol-2-yl)-2,5-diphenyltetrazolium bromide) test. Following treatment with blueberry extract and radiation, 104 cells were seeded in 100 µL of culture media per well and quadruplicated across 96-well plates. The culture media were aspirated and replaced with 5 mg/mL MTT solution after 24, 48, and 72 h of incubation. The MTT solution was carefully removed and replaced with DMSO (Sigma-Aldrich, Merck, Germany) after an additional 4 h of incubation. The absorbance was then measured at a wavelength of 570 nm. The following equation (Eq. 1) was used to calculate the cell viability [18].

1 $$\mathrm{Cell}\;\mathrm{vitatlity}\;\left(\%\right)=\frac{\mathrm{absorbance}\;\mathrm{of}\;\mathrm{experimental}\;\mathrm{group}}{\mathrm{absorbance}\;\mathrm{of}\;\mathrm{blank}\;\mathrm{control}\;\mathrm{group}}\times100$$

## Flow cytometry

The effects of radiation and blueberry extract on the distribution of the cell cycle and apoptosis in Raji cells were evaluated using flow cytometry. Raji cells were first cultivated using a full culture medium in each well of a 12-well plate at a density of 1 × 10^5^ cells/sample. Following treatment, the cells were exposed to different doses of blueberry extract (0, 600, 700, and 800 µg/ml) and were subsequently exposed to a single 2 Gy dose of radiation for 24 and 48 h. To detect apoptosis, Raji cells treated with radiation and blueberry extract were harvested at a density of 1 × 10^5^ cells/sample and then washed in a cold staining solution. After that, the cells received two cold PBS washes. Following that, the cells were analyzed using an apoptosis assay kit (BioLegend, USA) in compliance with the manufacturer’s instructions. In conclusion, each cell suspension was rinsed and resuspended in 100 µl of binding buffer before being supplemented with 5 µl of Annexin V-FITC conjugate and 5 µl of propidium iodide (PI) solution. The tubes were left in the dark for fifteen minutes at room temperature. The cells were immediately assessed using a flow cytometer. (BD Biosciences, San Jose, CA, USA) [24].

Raji cells treated with radiation and blueberry extract were harvested at a density of 1 × 10^5^ cells per sample to determine the cell cycle distribution, followed by cell collection and rinsing the cells with cold PBS. Then, the cells were examined using an apoptosis assay kit (BioLegend, USA) in accordance with the guidelines provided by the manufacturer. Propidium iodide (PI) was used in conjunction with 0.1% Triton X-100 and 0.1% sodium citrate to detect cell viability and death; Triton X-100 permeabilized the cell membranes, allowing PI to enter and bind to the DNA of non-viable cells, while sodium citrate stabilized the PI-DNA complex. The final cells were incubated for one hour at 4 °C. Using flow cytometry, the fluorescence intensity of labelled cells was determined in the FIL-1 channel [25].

### Analysis of gene expression

First, the cells were irradiated with a single 2 Gy dose for 24 and 48 h after being exposed to different doses of blueberry extract (0,600,700 and 800 µg/mL). After harvesting the cells, total RNA was extracted using the manufacturer’s technique using a Trizol reagent (Invitrogen, USA). Then, using 2 µg of total RNA from each sample, complementary DNA (cDNA) was synthesized according to the directions on the cDNA synthesis kit (Add Bio, Korea). RealQ Plus 2x Master Mix Green with High ROX (Ampliqon, Denmark) was employed to measure the *BCL-2*,* BAX*, and Xeroderma Pigmentosum Complementation Group A (*XPA)* gene mRNA expression levels.

The StepOnePlus Real-Time PCR Detection System (Thermo Fisher Scientific, USA) was employed to carry out the assays (primer sequences shown in Table 1). The reference gene in the study was *β-actin*. The target genes’ relative expression was assessed using the ΔΔCT method. The minimum information for publishing quantitative real-time PCR experiments (MIQE) requirements were adhered to in all real-time PCR techniques [17, 26].

**Table 1:** Primer sequences for RT-qPCR

Target gene	Primer sequences (5’→3’)	Product size
*BAX*	GGTCTTTTTCCGAGTGGCAGC (F) TGATCAGTTCCGGCACCTTGG (R)	131
*BCL-2*	CCTGTGGATGACTGAGTACCTGA (F) ACTGAGCAGAGTCTTCAGAGACA (R	145
*XPA*	AGCAAAGGAAGTCCGACAGG (F) CACACGCTGCTTCTTACTGCTC (R)	99
*β-actin*	CTGACGGCCAGGTCATCAC (F) CTGACGGCCAGGTCATCAC (R)	174

### Data analysis

Three-way and two-way analyses of variance (ANOVA, followed by Tukey’s test were performed to evaluate the significant differences between groups using Graph Pad Prism software 9.1.0 (San Diego, CA, USA). The results of p-value < 0.05 were considered statistically significant. The results were represented as mean ± SD of at least 3 determinations.

## Results and discussion

### Synergistic effect of blueberry extract and radiotherapy on the survival of Raji cells

**Fig. 1:**
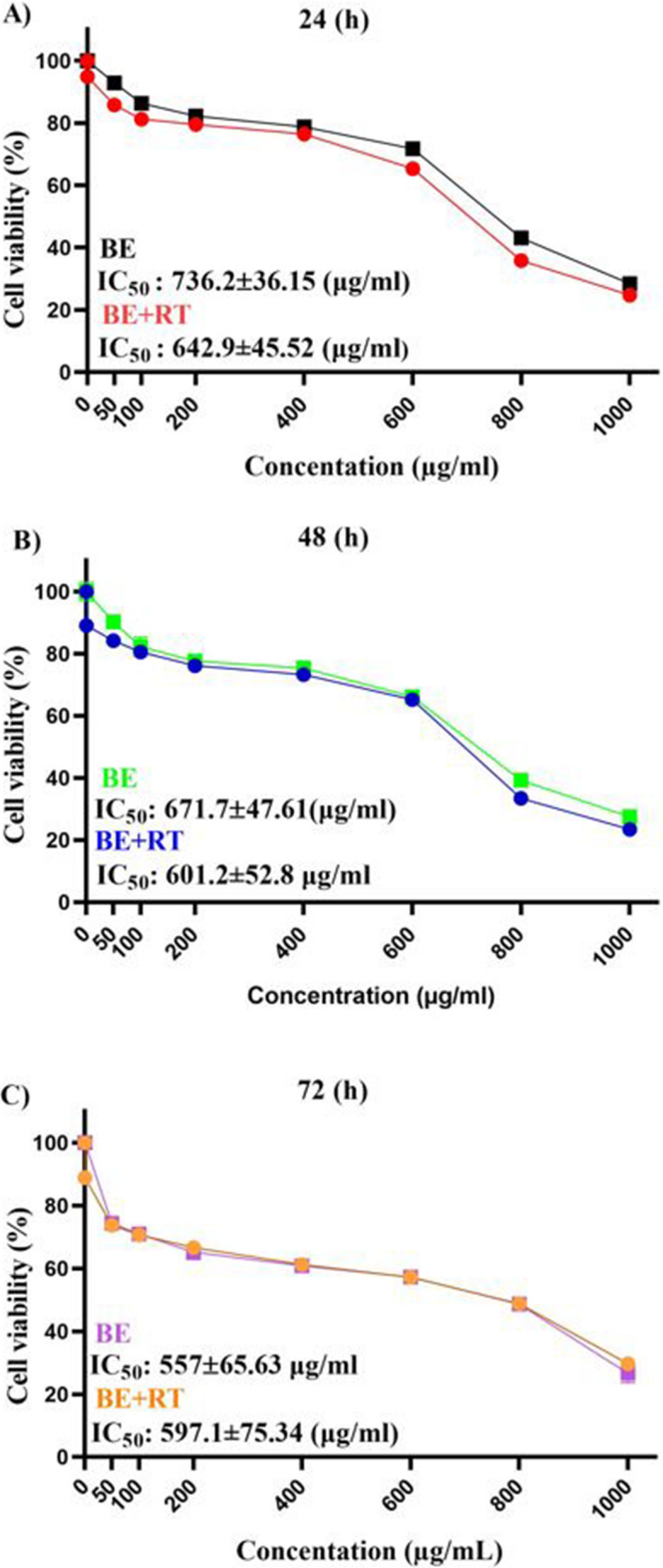
IC50 graphs (mean ± SE) for Raji cells treated with blueberry extract alone and in combination with radiotherapy. **A**): 24 h, **B**) 48 h, and **C**) 72 h

The MTT assay was utilized in this study to investigate the effects of combining blueberry extract with a single dose of 2 Gy radiation for 24, 48, and 72 h on the survival of Raji cells. Figure 1 displays the IC50 values for the various groups. All groups treated with blueberry extract combined with radiotherapy had lower IC50 values than groups treated with radiotherapy alone, except the group treated with blueberry extract for 72 h.

**Fig. 2:**
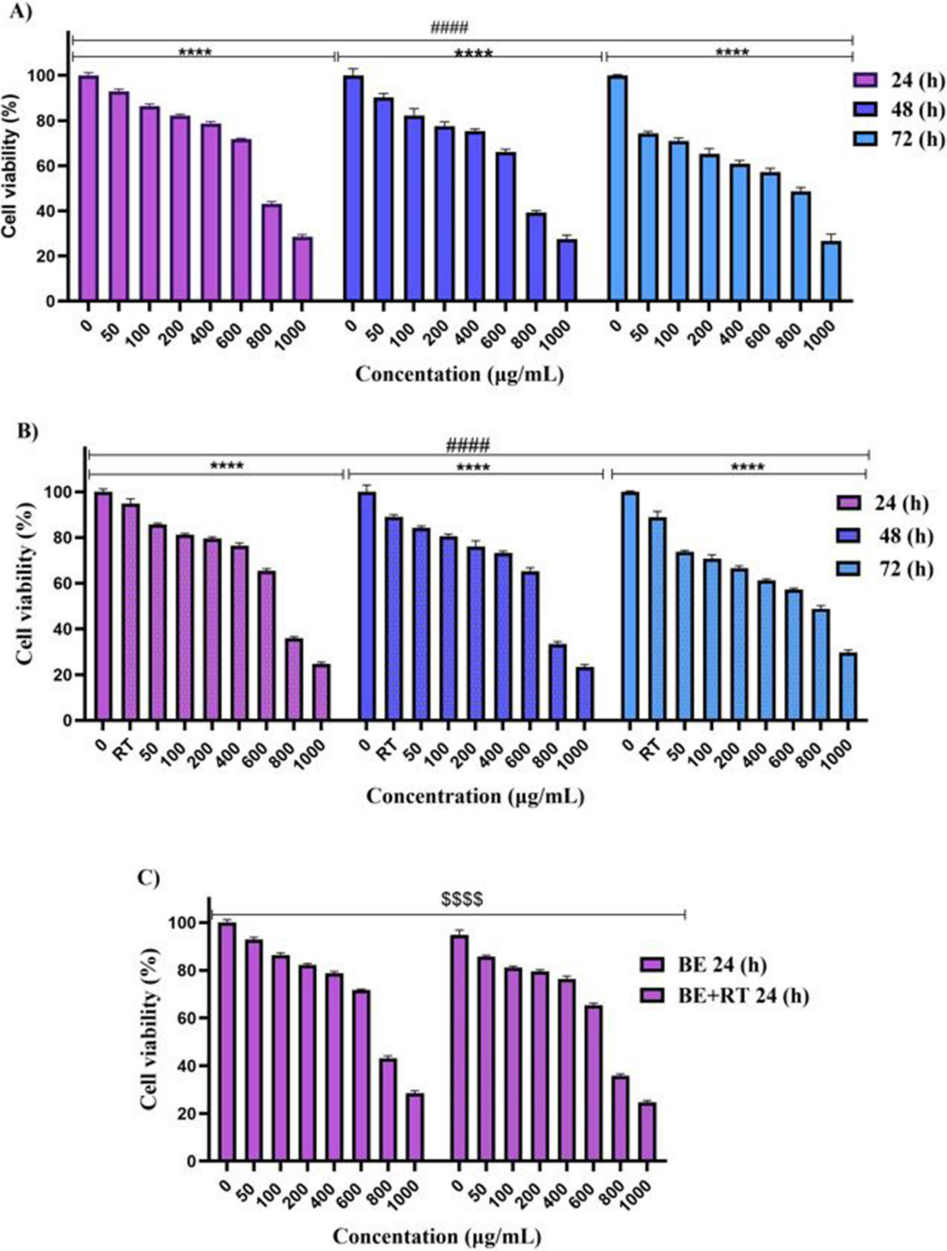

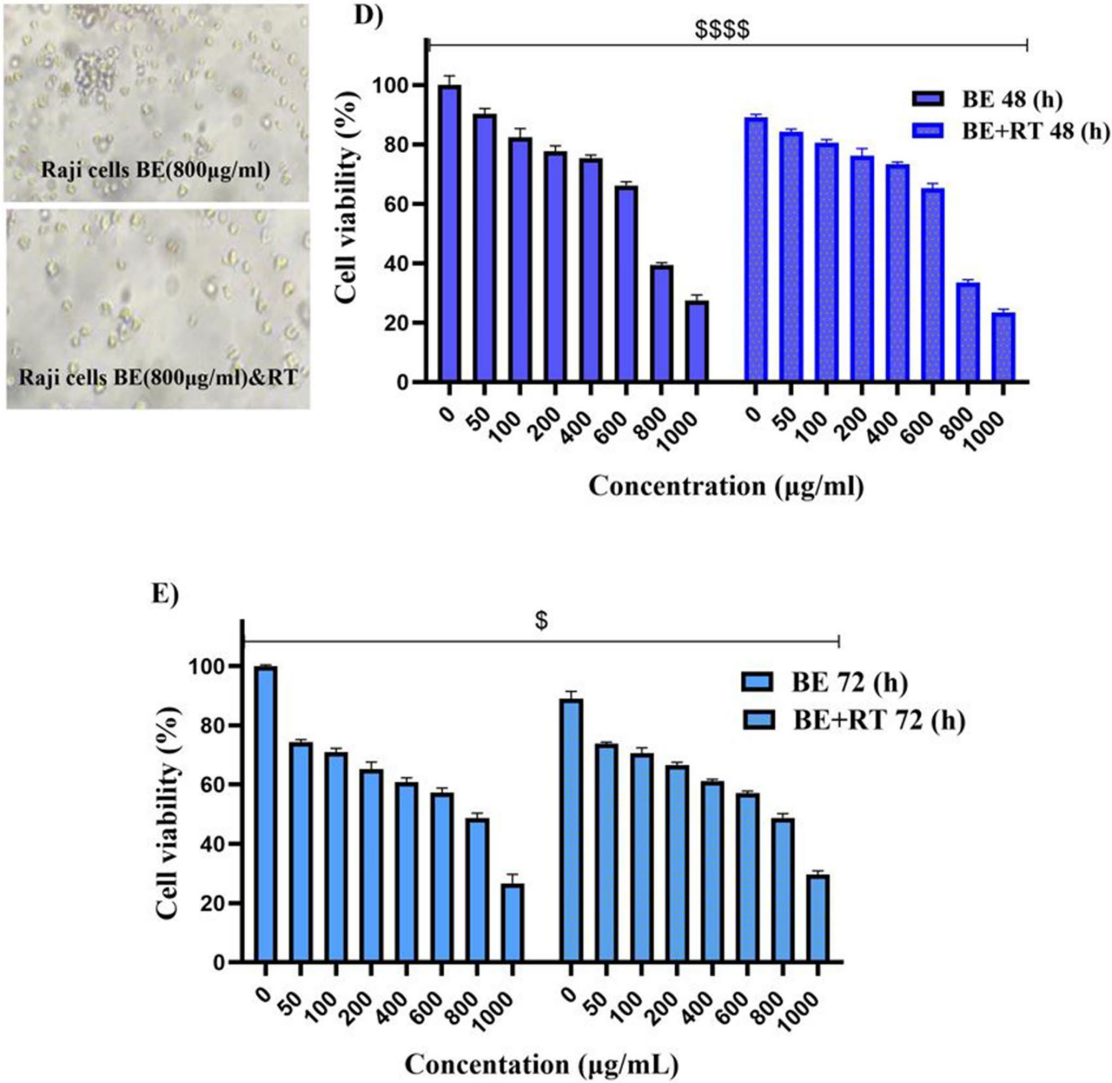
The results of the combined effect of blueberry extract with or without radiotherapy (**A, B**): Treatment with different concentrations of blueberry extract with (**B**) or without (**A**) a single dose of 2 Gy radiotherapy for 24, 48, and 72 h. (**C**, **D** and **E**): Comparison graph of the synergistic effect of blueberry on radiotherapy in 24 (**C**), 48 (**D**), and 72 (**E**) hours (*P* < 0.0001). (*) *P* < 0.05, (**) *P* < 0.01, (***) *P* < 0.001 vs., (****) *P* < 0.0001. Significant changes in the synergistic effect of blueberry on the radiotherapy group compared to the control group are represented by dollar signs. ($) *P* < 0.05, ($$) *P* < 0.01, ($$$) *P* < 0.001, () *P* < 0.0001. Significant changes in the effect of time in the treatment with blueberry extract with or without radiotherapy group compared to the control are indicated with hashtags. (#) *P* < 0.05, (##) *P* < 0.01, (###) *P* < 0.001, (####) *P* < 0.0001. (*n* = 4)

The findings demonstrated that blueberry extract exhibited a time- and dose-dependent decrease in cell viability compared to 0 µg/ml concentration (as control cells) (*P* < 0.0001). Furthermore, combined treatment of the extract with radiotherapy showed a synergistic effect, resulting in a significant reduction in cell viability compared to cells treated with radiotherapy alone (*P* < 0.0001) (Fig. 2). Comparing all the results demonstrated that blueberry extract had an improving effect on irradiation (*P* < 0.05) efficiency and that the combination treatment of blueberry extraction and radiation at a dose of 4 Gy reduced the proliferation of cervical cancer cells (SiHa).

### Enhanced apoptotic response in Raji cells with blueberry extract and radiotherapy combination

Raji cells were treated with concentrations near the IC50 value, along with one higher and one lower concentration (600,700,800 µg/ml), both with and without radiotherapy, over periods of 24 and 48 h. The 72-hour time point was excluded because the MTT results showed that the IC50 obtained with radiotherapy was lower compared to treatment with the extract alone. Annexin-V-FITC flow cytometry was utilized to demonstrate that this extract induces apoptosis of Raji cancer cells both alone and in combination with radiotherapy. In comparison to the control group, the results demonstrated that all groups of Raji cells treated with various amounts of blueberry extract induced apoptosis in a dose- and time-dependent manner during a period of 24 and 48 h (*P* < 0.0001). The cells treated with 800 µg/ml of blueberry have the highest rate of apoptosis (70.71%) after 48 h compared to control cells.

Additionally, the combined treatment (blueberry extract and radiotherapy) increased the apoptosis percentage in different groups compared to control cells. The rate improved significantly in cells treated with 800 µg/ml of BE and radiotherapy; after 48 h, it rose from 46.42% in control cells (only exposed to radiation) to 81.12% in the BE and radiotherapy-treated cells.

**Fig. 3:**
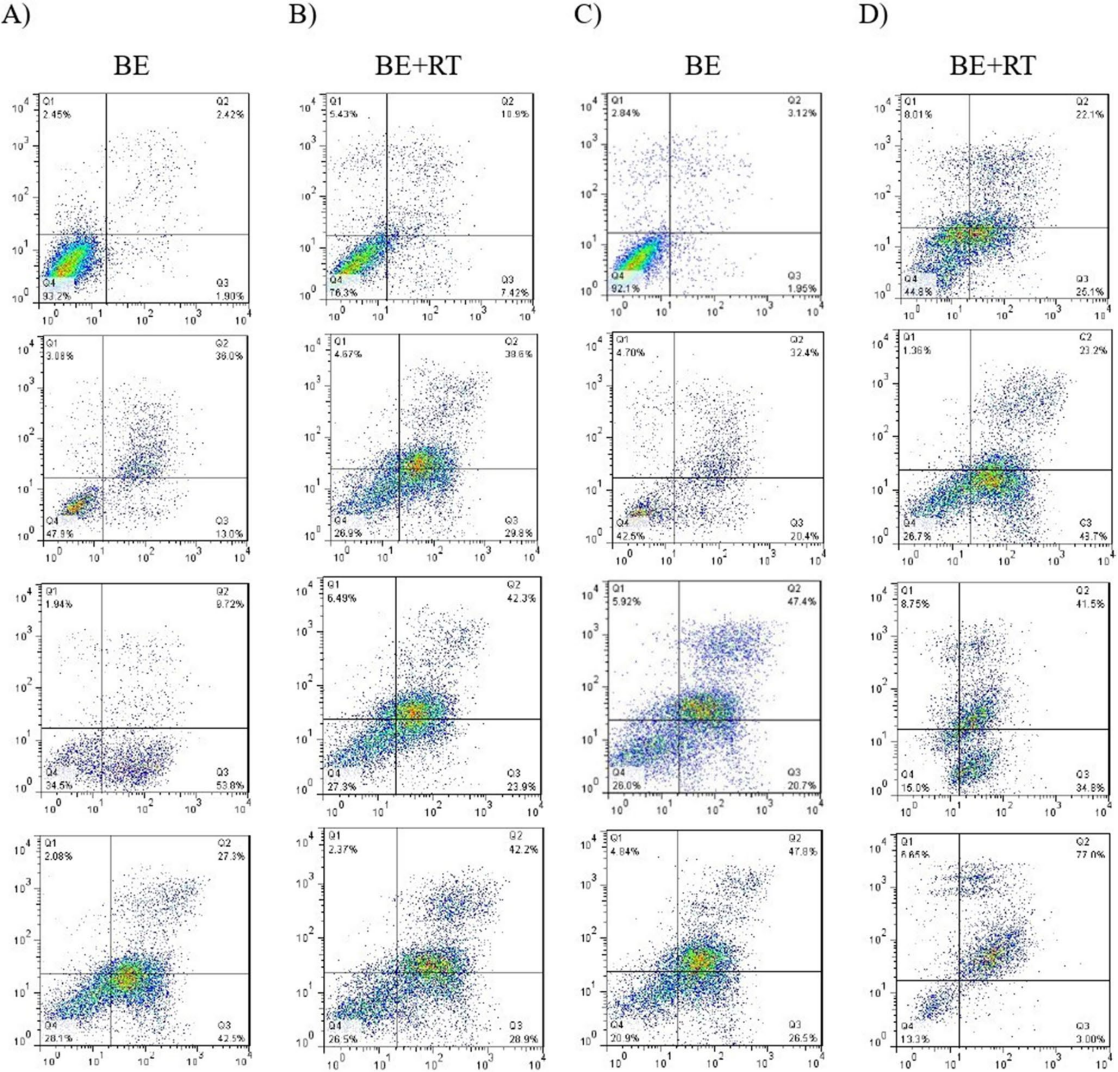

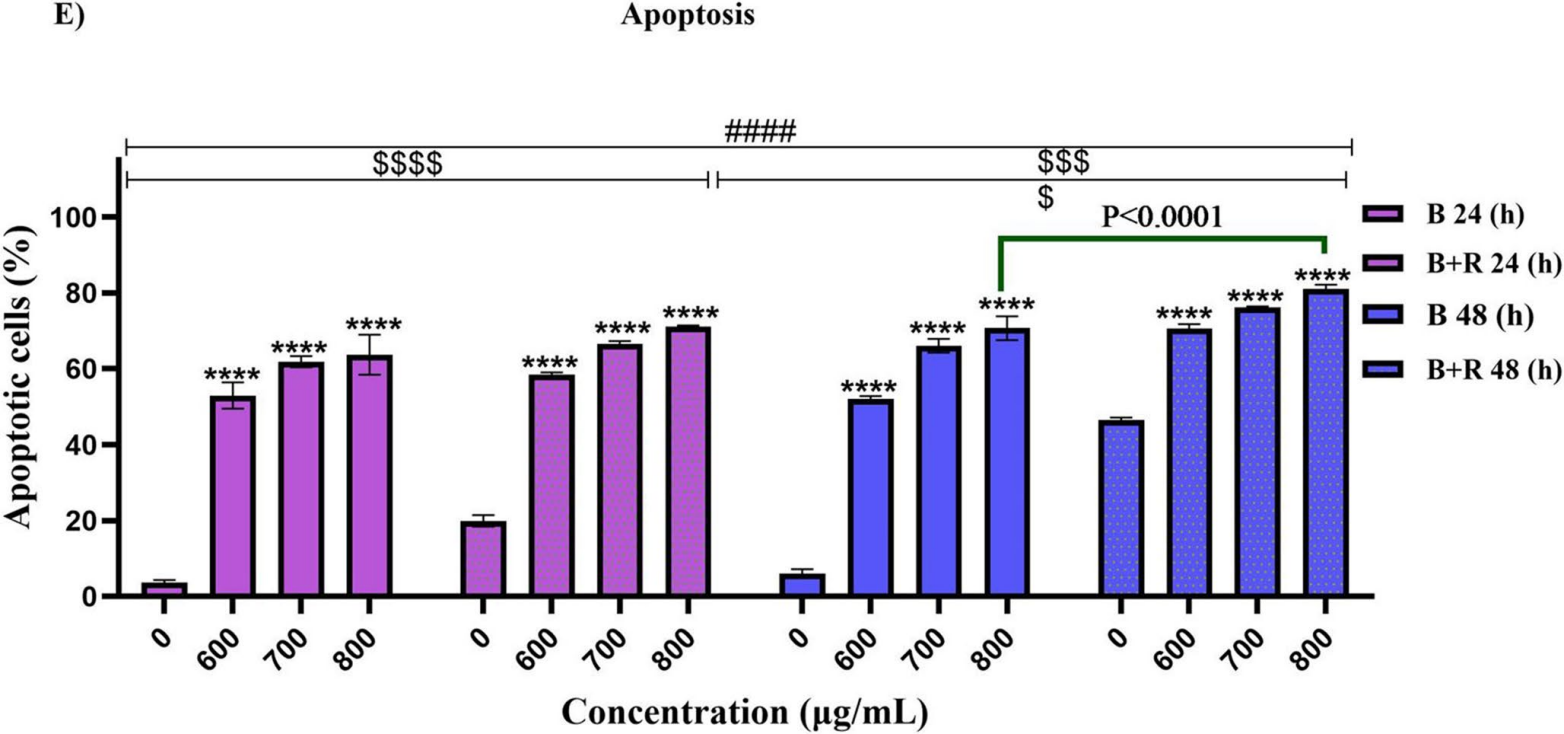
Influence of blueberry extract on apoptosis induction in Raji cells with (**B**, **D**) and without (**A**, **C**), flow cytometry analysis (**E**): Statistical analysis results in apoptosis rates. Significant changes in the impact of concentration in the blueberry extract group compared to the control are denoted with asterisks (*), *P* < 0.05. (**) *P* < 0.01. (***) *P* < 0.001 vs. (****) *P* < 0.0001. Significant changes in the synergistic effect of blueberry on the radiotherapy group compared to the control group are represented by dollar signs. ($) *P* < 0.05. ($$) *P* < 0.01. ($$$) *P* < 0.001. () *P* < 0.0001. Significant changes in the effect of time in the treatment with blueberry extract with or without radiotherapy group compared to the control are indicated with hashtags. (#) *P* < 0.05. (##) *P* < 0.01. (###) *P* < 0.001. (####) *P* < 0.0001 (*n* = 3)

This increase in apoptosis in cells treated with BE concurrently with RT, compared to cells that received only a 2 Gy dose of radiotherapy, demonstrates the radiosensitizer effect of this extract (*P* < 0.0001). This increase in apoptosis in Raji cells treated with blueberry extract with and without radiotherapy confirms the results of the MTT assay (Fig. 3).

Blueberry extract and its components have antioxidant, anti-inflammatory, and apoptosis-inducing effects on cancer cells [15, 25, 27]. According to research, the addition of blueberry extract components (specifically anthocyanin) to HeLa cancer cells increased apoptosis significantly, reaching 71.3% in cells treated with 800 µg/mL of the extract [25]. Additionally, the anthocyanidins in BE promoted dose- and time-dependent apoptosis in triple-negative breast cancer cells [15]. In another investigation, Kristoffer et al. [17] reported that blueberry extract increases apoptosis in cervical cancer cells while suppressing proliferation and improving the effectiveness of BE and RT.

### Enhanced Sub-G1 Cell Population Arrest in Raji Cells in Response to Blueberry Extract with or without Radiotherapy

Similar to the apoptotic stage, Raji cells were treated with BE at 600, 700, and 800 µg/ml concentrations for 24 and 48 h, with and without radiation therapy. Cell cycle analysis (Fig. 4) showed that treatment of Raji cells with blueberry extract alone or in combination with radiotherapy caused an arrest of the cells in the sub-G1 (*P* < 0.0001). Specifically, at a concentration of 800 µg/mL, the percentage of cells in this phase increased to 73.01% compared to control cells after 48 h. Additionally, treatment of cells with blueberry extract combined with radiotherapy caused cell cycle arrest in the Sub-G1 phase, indicating increased cell death with higher extract concentrations and longer treatment durations (*P* < 0.0001). Furthermore, at a concentration of 800 µg/mL and with radiotherapy at a 2 Gy dose, the percentage of cell death in control cells that received only radiotherapy increased from 46.91 to 79.73% after 48 h. These findings indicate the sensitizing effect of blueberry extract in combination with radiotherapy on cancer cells. Additionally, treatment with blueberry extract, either alone or in combination with radiotherapy, significantly reduced the number of cells in the G0/G1 phase (*P* < 0.0001) and showed a notable decrease in the S phase at concentrations ranging from 600 to 800 µg/mL with a 2 Gy radiotherapy dose (*P* < 0.0001). Moreover, a significant reduction in the G2/M phase was observed after 24 h of treatment with blueberry extract, both with and without radiotherapy, as well as after 48 h (*P* < 0.0001).

In a related study, Wang et al. demonstrated that administering Mv-3-gal, a component of blueberries, to hepatocellular carcinoma cells (Huh_7) at dosages of 50 and 100 µg/mL caused cell cycle arrest in the S phase. Cell cycle arrest in the G1 phase was the result of treatment with 200 µg/ml of Mv-3-gal, indicating the extract’s ability to inhibit the proliferation of Huh-7 cancer cells [16]. Additionally, treatment of MDA-MB-231 cells with anthocyanidin resulted in cell cycle arrest at the G2/M phase [15]. The previous studies demonstrated that combination therapy with resveratrol (plant polyphenols), either alone or alongside radiotherapy, increased SUB-G1 cells in the HELA, K-562, and IM-9 cell lines [21].

### Cell cycle

**Fig. 4:**
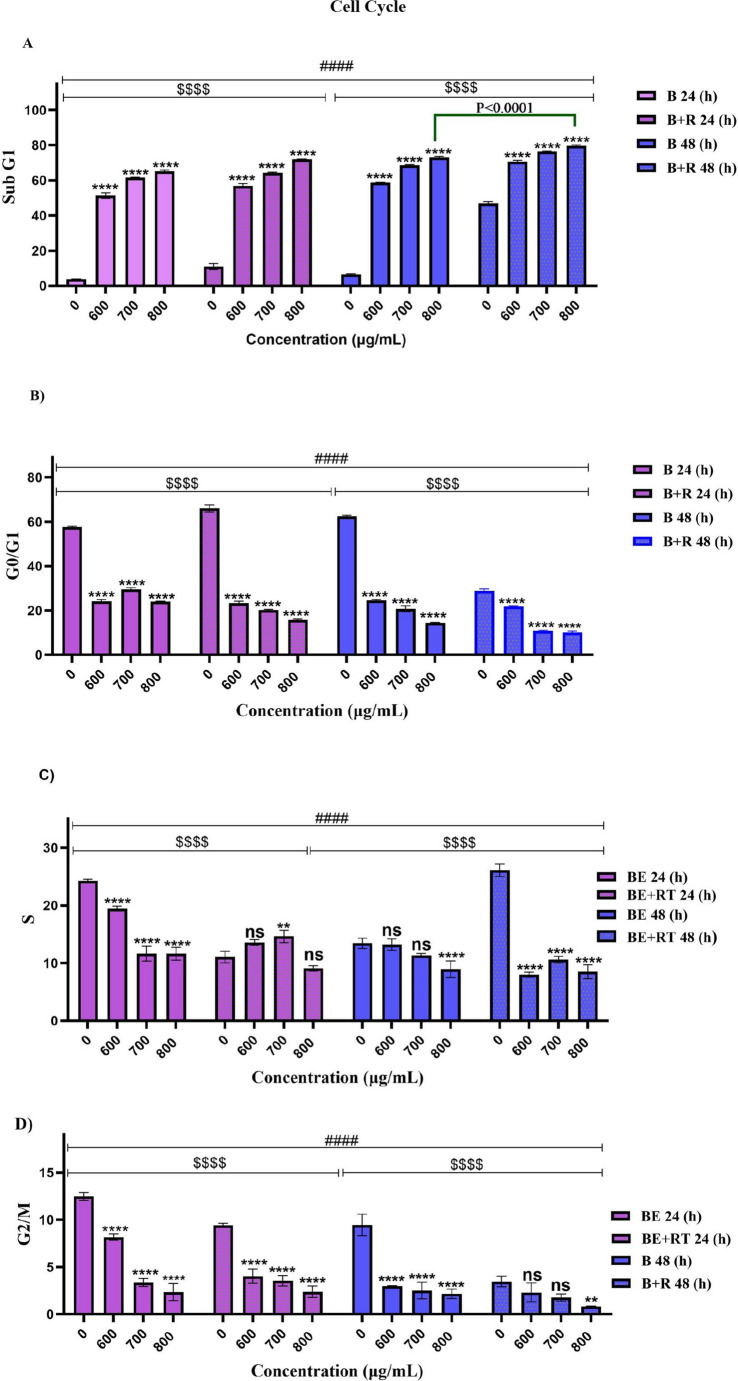
Cell cycle arrest by blueberry extract, with and without radiotherapy, flow cytometry analysis. (**A**, **B**, **C**, **D**). Significant changes in the impact of concentration in the blueberry extract group compared to the control are denoted with asterisks (*) *P* < 0.05. (**) *P* < 0.01. (***) *P* < 0.001 vs. (****) *P* < 0.0001. Significant changes in the synergistic effect of blueberry on the radiotherapy group compared to the control group are represented by dollar signs. ($) *P* < 0.05. ($$) *P* < 0.01. ($$$) *P* < 0.001. () *P* < 0.0001. Significant changes in the effect of time in the treatment with blueberry extract with or without radiotherapy group compared to the control are indicated with hashtags. (#) *P* < 0.05. (##) *P* < 0.01. (###) *P* < 0.001. (####) *P* < 0.0001. (*n* = 3)

### Effect of blueberry extract with and without radiotherapy on expression of BAX, XPA and BCL-2 in Raji cells

Following tests for cell cycle, apoptosis, and survival, Raji cells were treated with different blueberry concentrations (600, 700, and 800 µg/ml) and exposed to radiation therapy in order to evaluate gene expression. Results showed that treatment with different concentrations of blueberry extract led to dose and time-dependent upregulation of the apoptotic gene *BAX* in Raji cells compared to control cells in 24 and 48 h (*P* < 0.0001). Furthermore, concurrent therapy with blueberry extract and radiotherapy increased *BAX* levels in Raji cells compared to control cells in 24 and 48 h, and it showed a direct correlation with the extract concentration and treatment duration (*P* < 0.0001). Compared to cells treated with a single dose of 2 Gy radiotherapy alone, the combination of extract and radiotherapy synergistically affected *BAX* expression in 48 h (*P* < 0.001), confirming apoptosis induction by Annexin-V-FITC flow cytometry. Additionally, the blueberry extract (BE) in combination with radiotherapy reduced anti-apoptotic gene *BCL-2* expression in Raji cells compared to control cells in 24 and 48 h (*P* < 0.0001). This reduction was correlated with a reduction in dose and time (*P* < 0.001). Moreover, a decrease in anti-apoptotic *BCL-2* gene expression was observed in cells treated with a single dose of 2 Gy radiotherapy alone and in the cells treated with a combination of BE and radiotherapy (*P* < 0.001), demonstrating the radiosensitizing effect of the extract. The *BCL-2/BAX* ratio decreased with increasing extract concentration and treatment duration, particularly in cells receiving radiotherapy, indicating an enhanced propensity for apoptosis.

**Fig. 5:**
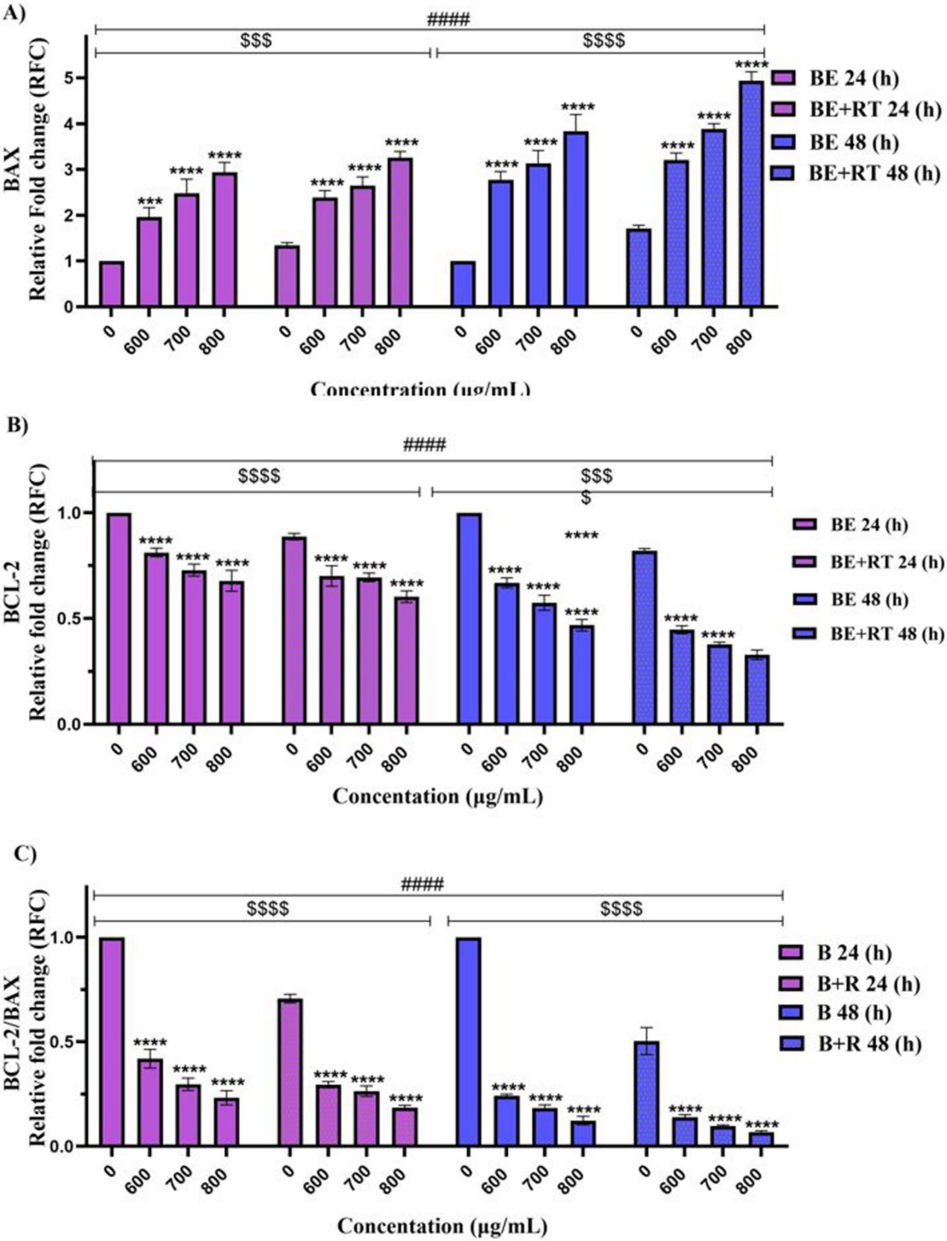

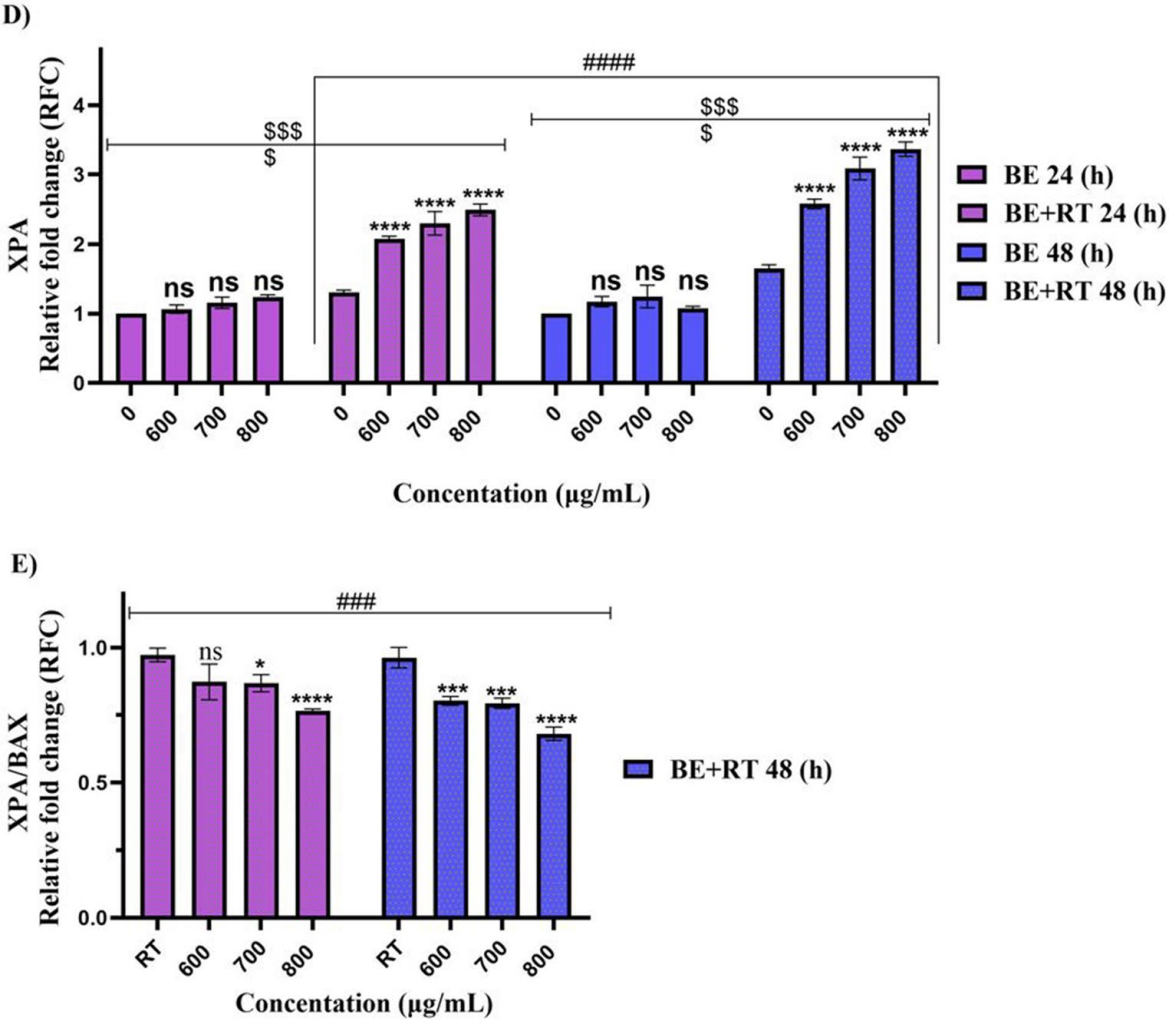
The effect of blueberry extract with and without radiotherapy on the expression of *BAX*,* XPA*, and *BCL-2* genes in Raji cells. Statistical analysis of qRT-PCR data: (**A**): Relative fold changes in *BAX* expression. (**B**): Relative fold changes in *BCL-2* expression. (**C**): *BCL-2/BAX* ratio D): Relative fold changes in *XPA* expression. E): *XPA/BAX* ratio. Significant changes in the impact of concentration in the blueberry extract group compared to the control are denoted with asterisks (*), *P* < 0.05. (**) *P* < 0.01. (***) *P* < 0.001 vs. (****) *P* < 0.0001. Significant changes in the synergistic effect of blueberry on the radiotherapy group compared to the control group are represented by dollar signs. ($) *P* < 0.05. ($$) *P* < 0.01. ($$$) *P* < 0.001. () *P* < 0.0001. Significant changes in the effect of time in the treatment with blueberry extract with or without radiotherapy group compared to the control are indicated with hashtags. (#) *P* < 0.05. (##) *P* < 0.01. (###) *P* < 0.001. (####) *P* < 0.0001. (*n* = 3)

In addition to *BAX* upregulation, blueberry extract with radiotherapy also increased expression of the DNA repair gene *XPA* in Raji cells. This increase was significant in cells treated with extract and radiotherapy compared to control cells (*P* < 0.0001) after 24 and 48 h. This was not the case when the extract alone was used to treat the Raji cells and compared to the control (*P* > 0.05). The elevation of *XPA* expression in cells treated with extract and radiotherapy compared to radiotherapy alone underscores the synergistic effect of the extract with radiotherapy in 24 and 48 h (*P* < 0.0001). The *XPA/BAX* ratio also declined dose- and time-dependently (*P* < 0.001) (Fig. 5).

The genes involved in the apoptosis process can be categorized into anti-apoptotic genes and pro-apoptotic genes [28, 29]. *BCL-2* is one of the anti-apoptotic genes that reside in the outer and inner membranes of mitochondria. A reduction in the expression of this gene can promote apoptosis [28]. On the other hand, *BAX* is one of the pro-apoptotic genes and an increase in its expression can promote apoptosis [29]. A study suggested that maintaining a balance in apoptotic and anti-apoptotic proteins is crucial in cancer cell growth [30]. Our study demonstrated that BE alone or in combination with RT increases the expression of the *BAX* gene and decreases the expression of *BCL-2*. Wu et al. [31] also showed that Luteolin, a type of flavonoid found in various vegetables and fruits such as blueberries, decreased *BCL-2* expression dose-dependently in adenocarcinoma cells (BGC-2, SGC-7901). In a similar study, it was reported that treatment of prostate cancer cells (LNCaP) with green tea extract rich in polyphenolic compounds decreased *BCL-2* expression and increased *BAX* gene expression [32]. According to the author, hepatocellular carcinoma cells (HepG2, SK-Hep-1) treated with IC50 and half IC50 concentrations of blueberry powder-enriched paste extracts in a dose-dependent manner exhibit a reduction in *BCL-2* expression in western blot analysis, indicating a drop in the *BCL-2/BAX* ratio [20]. Another study investigating the synergistic effect of resveratrol on radiation therapy in melanoma cancer cells revealed that while the combination treatment decreased *BCL-2* expression, *BAX* expression was unaffected [33]. This discrepancy between our study and previous studies may be due to differences in the use of different extract concentrations and cancer cell properties.

Radiotherapy aims to damage the DNA of cancer cells well enough so that it overcomes the ability of cells to repair itself, ultimately resulting in cell death. However, a number of cancer types are resistant to radiation due to factors like highly specialized DNA damage response mechanisms that allow for prompt damage detection and eventual repair. Therefore, a comprehensive understanding of these pathways is required to overcome biological radioresistance [34]. In addition, radiotherapy induces the production of ROS, which in turn activates various transcription factors, including AP-1, Sp1, NRF2, and p53. Notably, several genes involved in DNA repair possess redox-sensitive motifs within their promoter regions that bind these transcription factors. For instance, the promoters of the *XPA*,* XPB*,* XPC*, and *XPD* genes are crucial for nucleotide excision repair [35, 36]. The protein *XPA* is essential in the repair of DNA damage by nucleotide excision repair (NER), particularly under conditions like radiation. Protein *XPA* is a crucial part of a complex with Replication Protein A (RPA). When DNA damage is recognized, *XPA* and RPA bind to the damaged site, stabilizing and maintaining the region for appropriate DNA repair [37, 38]. Increased expression of *XPA* correlates with higher sensitivity of cancer cells to radiotherapy, suggesting its potential as a sensitizer in cancer treatment. The function of *XPA* in DNA repair has been the subject of numerous studies. According to a study, Bi2S3@BSA nanoparticle therapy enhanced radiosensitivity and *XPA* expression in MRC5 cells [26]. In another study, the increased *XPA* expression in treated cells indicates resistance mechanisms in glioblastoma cells, which play a complex role in cellular response to radiotherapy [37, 39]. The result of our study showed that when the blueberry extract was combined with a single 2 Gy radiotherapy, *XPA* expression in Raji cells increased in a dose- and time-dependent manner. Moreover, the *XPA/BAX* ratio showed a decreasing trend under these conditions, indicating an increase in apoptotic potential. The results of the study showed that blueberry extract inhibited cellular proliferation, induced apoptosis, and promoted cell death by increasing *BAX* expression and decreasing *BCL-2* expression. Treatment in combination with radiotherapy also sensitized the lymphoma cells to radiotherapy.

## Conclusion

In conclusion, both blueberry extract alone and in combination with radiotherapy showed an ability to inhibit cell proliferation and induce apoptosis in vitro. This is evidenced by its capacity to increase pro-apoptotic gene expression and reduce anti-apoptotic gene expression. These results emphasize the potential of plant compounds as effective agents for suppressing cancer cell growth and improving the radiosensitivity of cancer cells. This research revealed that the blueberry extract not only acts as a cancer cell growth inhibitor but also acts as a radiosensitizer in conjunction with radiotherapy, particularly in the context of non-Hodgkin lymphoma cells.

## Data Availability

The data will be available on request.

## References

[1] Chu Y, et al. The epidemiological patterns of non-Hodgkin lymphoma: global estimates of disease burden, risk factors, and temporal trends. Front Oncol. 2023;13:1059914. 37333805 10.3389/fonc.2023.1059914 PMC10272809

[2] Huang J, et al. Global burden, risk factors, and trends of non-Hodgkin lymphoma: a worldwide analysis of cancer registries. Cancer Med. 2024;13(5):e7056. 10.1002/cam4.7056 PMC10935876 38477498

[3] Yadav BS, Dey T. Radiotherapy dose de-escalation in patients with high grade non-Hodgkin lymphoma in a real-world clinical practice. Radiat Oncol J. 2023;41(4):237–47. 38185928 10.3857/roj.2023.00339 PMC10772589

[4] Denlinger N, Bond D, Jaglowski S. CAR T-cell therapy for B-cell lymphoma. Curr Probl Cancer. 2022;46(1):100826. 35012754 10.1016/j.currproblcancer.2021.100826 PMC9284423

[5] Crombie J, LaCasce A. The treatment of Burkitt lymphoma in adults. Blood. 2021;137(6):743–50. 33171490 10.1182/blood.2019004099

[6] Chevalier F. Counteracting radio-resistance using the optimization of radiotherapy. Int J Mol Sci. 2020;21(5):1767. 32150868 10.3390/ijms21051767 PMC7084332

[7] Barazzuol L, Coppes RP, van Luijk P. Prevention and treatment of radiotherapy-induced side effects. Mol Oncol. 2020;14(7):1538–54. 32521079 10.1002/1878-0261.12750 PMC7332214

[8] Zhang S, et al. Enhancement of recombinant myricetin on the radiosensitivity of lung cancer A549 and H1299 cells. Diagn Pathol. 2014;9:68. 24650056 10.1186/1746-1596-9-68 PMC3994494

[9] Gong L, et al. Application of radiosensitizers in cancer radiotherapy. Int J Nanomed. 2021;16:1083–102. 10.2147/IJN.S290438 PMC7886779 33603370

[10] Khan T, et al. Anticancer plants: a review of the active phytochemicals, applications in animal models, and regulatory aspects. Biomolecules. 2019;10(1):47. 31892257 10.3390/biom10010047 PMC7022400

[11] Kiernozek E, et al. Biological activity of extracts from differently produced blueberry fruits in inhibiting proliferation and inducing apoptosis of HT-29 cells. Foods. 2022;11(19):3011. 36230087 10.3390/foods11193011 PMC9563960

[12] Tobar-Bolaños G, et al. Blueberry juice: bioactive compounds, health impact, and concentration technologies-a review. J Food Sci. 2021;86(12):5062–77. 10.1111/1750-3841.15944 34716717

[13] Yang J, Pi C, Wang G. Inhibition of PI3K/Akt/mTOR pathway by apigenin induces apoptosis and autophagy in hepatocellular carcinoma cells. Biomed Pharmacother. 2018;103:699–707. 29680738 10.1016/j.biopha.2018.04.072

[14] Sapio L, et al. Targeting CREB in cancer therapy: a key candidate or one of many?? An update. Cancers. 2020;12(11):3166. 33126560 10.3390/cancers12113166 PMC7693618

[15] Aqil F, et al. Anthocyanidins inhibit growth and chemosensitize triple-negative breast cancer via the NF-κB signaling pathway. Cancers (Basel). 2021;13(24):6248. 10.3390/cancers13246248 PMC8699375 34944868

[16] Wang Y, et al. Blueberry malvidin-3-galactoside suppresses hepatocellular carcinoma by regulating apoptosis, proliferation, and metastasis pathways in vivo and in vitro. J Agric Food Chem. 2019;67(2):625–36. 30586992 10.1021/acs.jafc.8b06209

[17] Davidson KT, et al. Blueberry as a potential radiosensitizer for treating cervical cancer. Pathol Oncol Res. 2019;25(1):81–8. 28963664 10.1007/s12253-017-0319-y

[18] Zhao F, et al. The extraction and high antiproliferative effect of anthocyanin from gardenblue blueberry. Molecules. 2023;28(6):2850. 36985822 10.3390/molecules28062850 PMC10054926

[19] Wang E, et al. Antiproliferative and proapoptotic activities of anthocyanin and anthocyanidin extracts from blueberry fruits on B16-F10 melanoma cells. Food Nutr Res. 2017;61(1):1325308. 10.1080/16546628.2017.1325308 PMC5492086 28680383

[20] Xue B, et al. Inducing apoptosis in human hepatocellular carcinoma cell lines via Nrf2/HO-1 signalling pathway of blueberry and blackcurrant powder manipulated oat bran paste extracts. J Funct Foods. 2022;89:104967.

[21] Baatout S, et al. Enhanced radiation-induced apoptosis of cancer cell lines after treatment with Resveratrol. Int J Mol Med. 2004;13(6):895–902. 15138632

[22] Changizi V, Gharekhani V, Motavaseli E. Co-treatment with ginsenoside 20(S)-Rg3 and curcumin increases radiosensitivity of MDA-MB-231 cancer cell line. Iran J Med Sci. 2021;46(4):291–7. 34305241 10.30476/ijms.2020.83977.1334 PMC8288497

[23] Virdis P, et al. Antiproliferative and proapoptotic effects of Inula viscosa extract on Burkitt lymphoma cell line. Tumor Biol. 2020;42(2):1010428319901061. 10.1177/1010428319901061 32013807

[24] Ahmed RM, et al. Antitumoral properties of the pomegranate peel and blueberry extracts against tongue carcinoma (in vitro study). Saudi Dent J. 2023;35(8):985–95. 38107049 10.1016/j.sdentj.2023.07.021 PMC10724359

[25] Pan F, et al. Stability of blueberry anthocyanin, anthocyanidin and pyranoanthocyanidin pigments and their inhibitory effects and mechanisms in human cervical cancer HeLa cells. RSC Adv. 2019;9(19):10842–53. 35515294 10.1039/c9ra01772k PMC9062492

[26] Nasehi L, et al. Efficient induction of apoptosis in lung cancer cells using bismuth sulfide nanoparticles. Iran J Biotechnol. 2024;22(1):e3629. 38827339 10.30498/ijb.2024.385844.3629 PMC11139445

[27] Lin J, et al. Malvidin-3-galactoside from blueberry suppresses the growth and metastasis potential of hepatocellular carcinoma cell Huh-7 by regulating apoptosis and metastases pathways. Food Sci Hum Wellness. 2020;9(2):136–45.

[28] Saddam M, et al. Emerging biomarkers and potential therapeutics of the BCL-2 protein family: the apoptotic and anti-apoptotic context. Egypt J Med Hum Genet. 2024;25(1):12.

[29] Ghasemi A, et al. Evaluation of BAX and BCL-2 gene expression and apoptosis induction in acute lymphoblastic leukemia cell line CCRFCEM after high-dose prednisolone treatment. Asian Pac J Cancer Prev. 2018;19(8):2319–23. 10.22034/APJCP.2018.19.8.2319 PMC6171400 30141309

[30] Fatemizadeh M, et al. Apoptosis induction, cell cycle arrest and anti-cancer potential of Tamoxifen-Curcumin loaded niosomes against MCF-7 cancer cells. Iran J Pathol. 2022;17(2):183–90. 35463725 10.30699/IJP.2022.124340.2356 PMC9013861

[31] Wu H, et al. Luteolin induces apoptosis by up-regulating miR-34a in human gastric cancer cells. Technol Cancer Res Treat. 2015;14(6):747–55. 24988056 10.7785/tcrt.2012.500434

[32] Safari F, et al. Antitumor activities of green tea by Up-regulation of miR-181a expression in LNCaP cells using 3D cell culture model. Avicenna J Med Biotechnol. 2022;14(1):89–94. 35509367 10.18502/ajmb.v14i1.8174 PMC9017474

[33] Fang Y, et al. A potential role for resveratrol as a radiation sensitizer for melanoma treatment. J Surg Res. 2013;183(2):645–53. 23522452 10.1016/j.jss.2013.02.037

[34] Melia E, Parsons JL. DNA damage and repair dependencies of ionising radiation modalities. Biosci Rep. 2023;43(10):BSR20222586. 37695845 10.1042/BSR20222586 PMC10548165

[35] Borszéková Pulzová L, Ward TA, Chovanec M. XPA: DNA repair protein of significant clinical importance. Int J Mol Sci. 2020;21(6):2182. 32235701 10.3390/ijms21062182 PMC7139726

[36] Hellweg C, et al. Transcription factors in the cellular response to charged particle exposure. Front Oncol. 2016;6:61. 27047795 10.3389/fonc.2016.00061 PMC4800317

[37] Borszéková Pulzová L, Ward TA, Chovanec M. XPA: DNA repair protein of significant clinical importance. Int J Mol Sci. 2020. 10.3390/ijms21062182. 10.3390/ijms21062182 PMC7139726 32235701

[38] Schärer OD. Nucleotide excision repair in eukaryotes. Cold Spring Harb Perspect Biol. 2013;5(10):a012609. 24086042 10.1101/cshperspect.a012609 PMC3783044

[39] Todorovic V, et al. Mechanisms of different response to ionizing irradiation in isogenic head and neck cancer cell lines. Radiat Oncol. 2019;14(1):214. 31775835 10.1186/s13014-019-1418-6 PMC6882348

